# Personal GP continuity improves healthcare outcomes in primary care populations: a systematic review

**DOI:** 10.3399/BJGP.2024.0568

**Published:** 2025-07-14

**Authors:** Sven Göran Engström, Malin André, Eva Arvidsson, Carl Johan Östgren, Margareta Troein, Lars Borgquist

**Affiliations:** 1 Futurum Academy of Health and Care, Region Jönköping County, Sweden; 2 Department of Public Health and Caring Sciences, Family Medicine and Preventive Medicine, Uppsala University, Uppsala, Sweden; 3 Department of Health, Medicine and Caring Sciences, Linköping University, Linköping, Sweden; 4 Department of Clinical Sciences, Faculty of Medicine, Lund University, Malmö, Sweden

**Keywords:** continuity of patient care, outcome assessment, health care, primary health care, systematic review

## Abstract

**Background:**

Personal continuity is a hallmark for GPs but there is insufficient evidence to support its benefits in ordinary primary care populations.

**Aim:**

To investigate the effects of GP personal continuity on the healthcare outcomes of primary care populations.

**Design and setting:**

Systematic review of quantitative studies investigating associations between personal continuity of care and outcomes such as mortality and healthcare utilisation.

**Method:**

Embase, PubMed, Scopus, and Web of Science were searched for studies published between 1 January 2000 and 31 October 2023. Owing to study heterogeneity the synthesis was conducted narratively; study results were summarised and expressed as having higher (compared with lower) continuity of care. Certainty of each summarised result was assessed using the GRADE framework.

**Results:**

Out of 5792 unique references, 18 studies were included in the final analyses. The outcomes were grouped into three categories of summarised outcomes. Higher (when compared with lower) personal continuity with a GP/family physician probably prevents premature mortality (moderate certainty: four studies, 5 638 305 participants), probably reduces the risk of admission to hospital (moderate certainty: 11 studies, 13 642 684 participants), and probably lowers risk of emergency department visits (moderate certainty: seven studies, 3 855 487 participants).

**Conclusion:**

Higher, compared with lower, continuity in the relationship between GP and patients in primary care populations is associated with reduced mortality, admissions to hospital, and emergency department visits. Relatively small improvements in personal continuity, which may be achieved in most practices, significantly reduce healthcare consumption, and thus may have an impact on access to care, which has implications for healthcare policy.

## Introduction

Continuity of care is widely considered to be an essential component of primary health care. At least three aspects are discussed in the literature: informational (access to data on earlier care and personal circumstances), management (timely provision of health care according to guidelines), and relational (an ongoing relationship between patient and physician, resulting in cumulative knowledge of the patient as a person).^1^ Relational continuity may exist with a single GP (also known as a family physician but referred to in this review as a GP), a practice (site continuity), or teams within a practice.

Personal continuity is a prerequisite for a relationship to arise, and has been linked to improvements in various outcomes for patients and healthcare systems. Several systematic reviews have found that personal continuity between the patient and physician is associated with reduced mortality, hospital admissions, emergency department visits, and costs in the health system.^2–7^ The studies included in these reviews, however, also address personal continuity in healthcare settings other than primary care, and patient populations selected for certain diagnoses or age groups. In addition, the physicians often represented other medical specialities than GP.1

**How this fits in**

Several systematic reviews have found that personal continuity between the patient and physician is associated with reduced mortality, hospital admissions, emergency department visits, and costs in the health system. In this review, focusing on personal continuity between GPs and patients in ordinary primary care populations, it was found that even relatively small improvements in personal GP continuity resulted in significant reductions in admissions and emergency department visits. Such improvements can probably be achieved in most practices.

The aim of this systematic review was therefore to investigate the effects of personal continuity between patients and GPs on the health outcomes of patients in ordinary primary care populations.

## Method

### Protocol

Prior to commencing this review, a study protocol was developed and registered with PROSPERO (reference number: CRD42023476099).

### Inclusion criteria and search strategy

A systematic literature review that adhered to the PRISMA reporting guidelines was conducted.^8^ The inclusion criteria were formulated using the patient, intervention, comparison, outcome (PICO)/patient, exposure, comparison, outcome (PECO) structure.^9^ The population had to be an ordinary primary care population, and not be restricted by diagnosis or age. As care of older adults is an important part of primary care, this review included studies that focused on older people.

**Figure 1.**
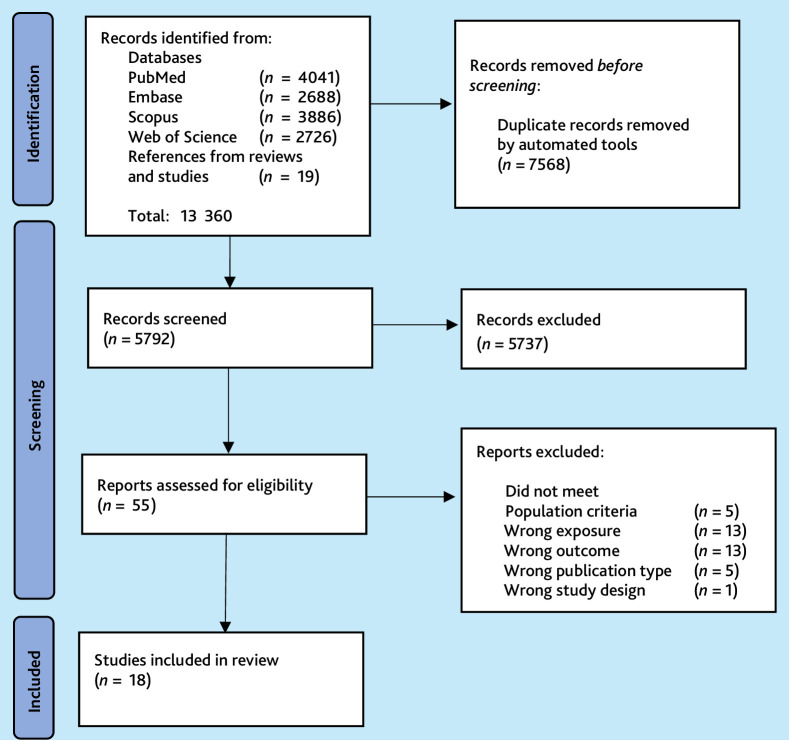
A PRISMA flow chart of the searched and included studies.

The exposure had to be personal continuity of care of the patients by a primary care physician who was a specialist GP, and quantified by an established continuity index or measure of duration, density, or discontinuation of care. The exposure should have been present for ≥12 months. Comparisons of outcomes for different levels of personal continuity (from low to high) should have been performed and reported. Included studies had to be published in a peer-reviewed journal between 1 January 2000 and 31 October 2023. The exclusion criteria and the number of excluded studies are shown in Figure 1.

The search strategy was developed by an information specialist with the assistance of the authors. Blocks of search terms about primary health care, physicians, and the exposure continuity/discontinuity of patient care were used in subject headings and titles and abstracts. The search was restricted to articles in English, Danish, Norwegian, or Swedish, and studies that had been conducted in countries with a primary care system consisting of GPs with specialist training times of ≥36 months. The searches in Embase, PubMed, Scopus, and Web of Science were performed in November 2023. Grey literature, books, and conference abstracts were not considered, see full search strategy in Supplementary Information S1.

Supplementary Information

### Assessment of relevance and quality

Screening of titles and abstracts to determine whether they fulfilled the inclusion criteria was performed independently by two researchers (the first author and the senior author) using the Rayyan web application.^10^ Disagreements regarding inclusion were resolved through discussion, although 55 studies had to be read in full in order to evaluate them for inclusion.

Four researchers (the first author, second author, third author, and senior author) independently read the 55 articles in full, and rated all with regard to their fulfilling the set inclusion criteria. The assessments of each study were then discussed; where assessments differed, the article was further reviewed.

To assess risk of bias, the ROBINS-I tool^11^ was used for assessing risk of bias in non-randomised studies of interventions.

### Data analysis, synthesis, and rating of the certainty of evidence

Data were extracted by one researcher (the first author) and checked for correctness by two others. Data extracted included study type, the country where the study was performed, study population, study period, study participants’ age, measurement of continuity, type of analysis, confounders, and main results/summary statistics for the outcomes.

The characteristics of the included studies were diverse, which precluded meta-analyses. Instead, results from studies relating to the chosen outcomes were synthesised without meta-analysis. The overall result for each category of outcome was formulated as a summarised result regarding effect. A decision was taken to describe the effects of personal continuity as percentage changes.

The GRADE framework was used to rate the certainty of evidence for each statement of summarised results as high, moderate, low, or very low.^12^

## Results

After removal of duplicate results, 5792 unique references were identified, and 55 articles were read in full. Eighteen articles fulfilled the inclusion criteria; these were all based on observational data and were mainly retrospective cohort studies. The identification and selection of the included studies is shown in Figure 1. A summary of characteristics of the included studies is shown in Supplementary Table S1.

The included studies were performed in Australia,^13^ Canada,^14–17^ France,^18^ Germany,^19^ England,^20,21^ Norway,^22–24^ Sweden,^25,26^ the Netherlands,^27^ and the US.^28–30^ The reported results are based on analyses of >15 million patients.

**Figure 2.**
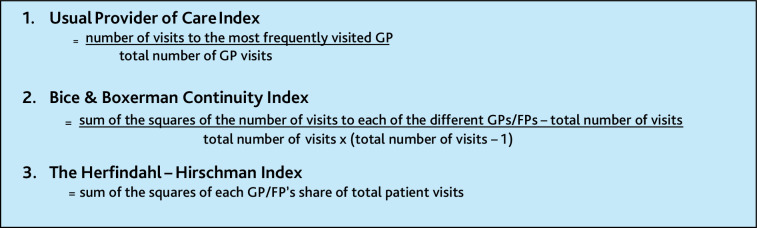
The three methods of measuring personal continuity. FP = family physician.

The continuity measures had two main starting points: either the number of years the patient had attended the same GP,^22,24^ or the proportion of the patients' consultations during the period of the study that had been conducted by the same GP.^13–21,23,25–30^ These proportions were measured using three different methods across the studies: the Usual Provider of Care Index (UPC), Bice and Boxerman Continuity Index (B&B), and Herfindahl–Hirschman Index (HH) (Figure 2).^31^ Where a study used >1 index, a decision was taken to present B&B, which, unlike UPC, also considers the number of different doctors seen by each patient, and then UPC. The measurements in the included studies applied per individual patient, while average values for all patients were used in the analyses.

The reported outcomes allowed categorisation into the following summarised outcomes: mortality,^18,23,24,27^ admission to hospitals,^13,17,19–24,28–30^ and emergency department visits.^14–16,25,26,29,30^

Below, the results for the different outcomes are presented, as are brief descriptions of the studies included in each outcome category. Table 1 provides a summary of the results and the assessments of certainty of evidence. In the GRADE rating of evidence five domains are used.

- Confounding bias: the authors, for all outcomes, reduced the level of certainity by1.0 because of the risks of bias.
- Imprecision: the studies include millions of patients and outcome events.
- Inconsistency: the outcomes in the studies went in the same direction, except that in one study,^13^ for one of the two comparisons of levels of continuity the results pointed in the opposite direction, and another study^15^ found no correlations.
- Indirectness: the continuity measures were well established. The studies reflected well the ordinary primary care populations and GPs.
- Publication bias: personal continuity is not an area where companies or others have financial interests.

Thus, the authors did not consider it appropriate to make deductions for the last four domains in GRADE.

**Table 1.** Summarised results and evidence ratings according to GRADE

Outcome	Studies, *n* (participants, *n*)	Summarised result	Certainty of evidence assessed according to GRADE	Reasons for reduced certainty of the evidence
Mortality	4 (5 638 305)	Higher (compared with lower) personal continuity between patients and GP probably reduces premature mortality by 10%–15%	Moderate	Risk of bias −1 Imprecision 0 Inconsistency 0 Indirectness 0 Publication bias 0
Admissions to hospital	11 (13 642 684)	Higher (compared with lower) personal continuity between patients and GP probably lowers the risk of future admission to hospital by 10%–15%	Moderate	Risk of bias −1 Imprecision 0 Inconsistency 0 Indirectness 0 Publication bias 0
Emergency department visits	7 (3 855 487)	Higher (compared with lower) personal continuity between patients and GP probably lowers the risk of emergency department visits by 10%–20%	Moderate	Risk of bias −1 Imprecision 0 Inconsistency 0 Indirectness 0 Publication bias 0

**Figure 3.**
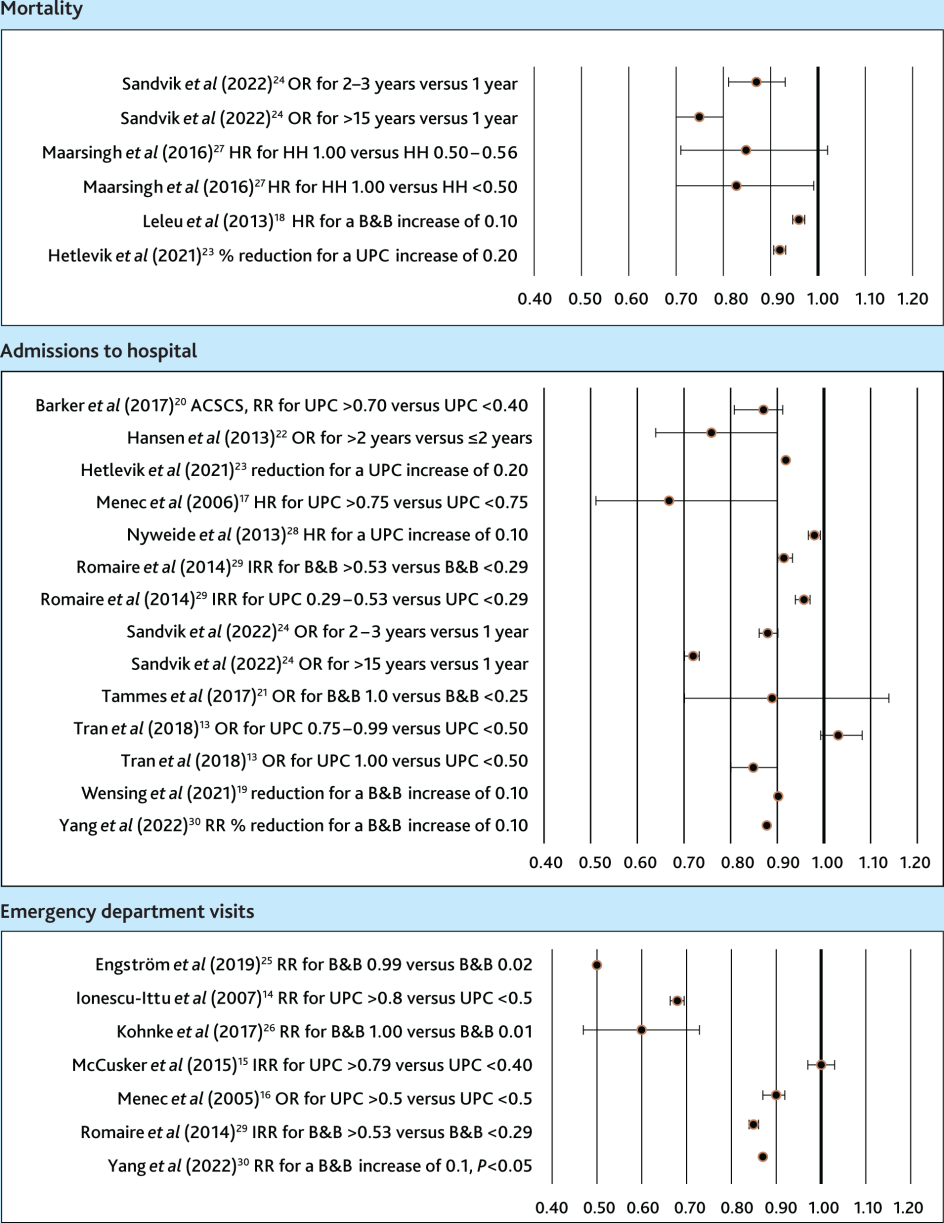
Forest plots of the effects of personal continuity between GPs and their patients on mortality, admissions to hospital, and emergency department visits. The studies of effects on outcomes compared high and low continuity using different measures of association, including odds ratio (OR), hazard ratio (HR), risk ratio (RR), and incidence rate ratio (IRR). Some studies used percentage reductions of outcomes. For each study, information is given in the following order: author, measure of association, measure of continuity, and the levels compared. In some of the studies lower continuity was compared with higher continuity;^14,21,26,28^ for these, the values have been inverted. Horizontal lines indicate confidence intervals. ACSC = ambulatory care sensitive condition. B&B = Bice and Boxerman Index. HH = Herfindal–Hirschman Index. UPC = Usual Provider of Care Index.

In Figure 3, forest diagrams illustrate the results of the different studies across the different indices and models used to assess associations.

### Mortality

Four studies^18,23,24,27^ investigated the association between personal continuity of care and mortality across 5 638 305 patients. Sandvik *et al* ^24^ reported that, compared with 1-year relationships between patients and registered GPs, the odds ratio (OR) for death decreased gradually with longer relationships: from 0.92 (95% confidence interval [CI] = 0.86 to 0.98) after 2–3 years to 0.75 (95% CI = 0.70 to 0.80) after >15 years. Hetlevik *et al* ^23^ concluded that when the UPC index increased by 0.2, mortality fell by 8.3% (*P*<0.001). Leleu *et al* ^18^ found that an increase in the B&B index of 0.1 was associated with a decreased likelihood of death (hazard ratio 0.96, 95% CI = 0.95 to 0.96). Maarsingh *et al* ^27^ used the HH index as a measure of continuity, and found that participants in the lowest continuity category showed significantly greater mortality than those in the highest category (hazard ratio 1.20, 95% CI = 1.01 to 1.42).

The overall result for the outcome mortality was: higher for low personal continuity between patients and GPs; better personal continuity probably reduces premature mortality by 10%–15%. The certainty of the evidence was moderate according to GRADE.

### Admissions to hospital

Eleven studies^13,17,19–24,28–30^ with a total of 13 642 684 participants investigated the effect of personal continuity of care on risk of future admission to hospital. The analytical approaches used varied; however, all of the studies but one^13^ found that higher personal continuity was clearly associated with a lower frequency of admissions to hospital.

The overall result for the outcome admission to hospital was: higher for low personal continuity between patients and GPs; better personal continuity probably lowers the risk of future admission to hospital by 10%–15%. The certainty of the evidence was moderate according to GRADE.

### Emergency department visits

Seven studies^14–16,25,26,29,30^ with a total of 3 855 487 participants investigated the effect of personal continuity of care on the risk of future emergency department visits. All studies used continuity measures that consider the proportion of visits to the same GP. One study^15^ found that personal continuity had no effect on emergency department visits. The other six found that higher values for the UPC and/or B&B indices were associated with fewer emergency department visits.

The overall result for the outcome emergency department visits was: higher for low personal continuity between patients and GPs; better personal continuity probably lowers the risk of emergency department visits by 10%–20%. The certainty of the evidence was moderate according to GRADE.

## Discussion

### Summary

In this systematic review, 16 of 18 studies from various countries and healthcare systems showed that increased personal continuity was related to improved healthcare outcomes. Two studies^13,15^ found no association between continuity and healthcare use. Hence, the results suggest that higher personal continuity between patients in ordinary primary care populations and GPs may prevent premature deaths, and lowers the risk of admission to hospital and emergency department visits. The certainty of the evidence was, according to the rating using GRADE, assessed as moderate.

To the authors’ best knowledge, this is the first systematic review to strictly address the effects of personal continuity between patients in ordinary primary care populations and GPs that did not restrict the scope in terms of diagnoses or demographics.

### Strengths and weaknesses

Most of the studies included very large numbers of patients (total roughly 15 million) and events (2.5 million). All of the studies adjusted for some measure of health, such as the number of chronic diagnoses or a disease score (such as the Charlson Score^32^ and Adjusted Clinical Groups^33^), and for sociodemographics.

Another strength of the study is a high consistency in the finding of positive outcomes for high personal continuity of care, in spite of the fact that different kinds of continuity measures were used in the various studies. Most studies also demonstrate dose-dependent effects of personal continuity.

This study has some important limitations. All studies are observational studies, hence cause-and-effect relationships cannot be claimed. All of the included studies had non-randomised designs, which may imply an increased risk of bias owing to confounding. There are also difficulties in separating continuity effects from other practice characteristics, that is, a high continuity may reflect practices with better staffing or organisational systems. The review spans multiple countries and thus variations in healthcare systems and differences in care models, funding mechanisms, and physician roles might affect the generalisability of the findings. It should be acknowledged that there might be a publication bias toward positive findings about continuity effects. The confounders of particular concern are the health status and morbidity of the study participants, which can affect both the need for and access to personal continuity of care, and the outcomes studied. Reverse causality must be considered, but is countered by the fact that eight of the studies had designs that ensured no overlap between the time of continuity measurement and the period during which outcomes were measured.

In the absence of true randomised experimental settings, however, observational studies that control rigorously for confounding factors and have designs that are intended to limit the impact of reverse causality are the best evidence available.

### Comparison with existing literature

The results of this review are in line with those of earlier studies, which did not focus specifically on general practice populations and GPs. Recent reviews of studies concerning other populations have found that personal continuity is associated with reduced mortality, emergency department visits, and admissions to hospital.^3,5,6^

The possible mechanisms for the effects of personal continuity on healthcare outcomes is analysed in a review by Sidaway-Lee *et al*.^34^ Repeated contact increases the patient’s trust in the doctor, adherence to prescriptions, confidence in treatment, and satisfaction.^35^ The feeling of patients that they ‘know’ their GP has been associated with enablement.^36^ One of the studies investigated in the review found that eight consultations with the same GP were required for there to be a 50% probability of the patient feeling that the working relationship was ‘deep’.^37^ Repeated contact increases the physician’s knowledge of the patient, which may result in care that is better tailored to the needs of the patient as a person,^38^ and improve patient–physician communication.^39^ Personal continuity increases the sense of responsibility^40^ and empathy^41^ that GPs have for their patients. When a GP sees a patient repeatedly, it is possible to compare their current state with how they normally look and behave.

Doctor–patient relationships may also have important adverse effects. For example, increased empathy could make a GP more reluctant to bring up difficult topics, which may lead to delayed diagnoses. Time is important. Evidence suggests that the duration of the patient–GP relationship has more significance for the patient’s perception of care than the share of visits to the same physician.^39,42^ The importance of long relationships is underlined by the results of the study conducted by Sandvik *et al*,^24^ included in the current review. This study compared 15-year patient–GP relationships with 10-year ones, and found a greater reduction in mortality, emergency admissions, and use of out-of-hours care for the former.

### Implications for research and practice

A crucial factor in the positive impact of personal continuity in primary care on healthcare outcomes is the relationship between patient and doctor. If such a relationship is established and patients and GPs know each other, the negative impact of an occasional visit to another GP can be minimal.

A growing body of evidence suggests that personal continuity is a key factor in and perhaps mediator of quality of care, and so healthcare authorities in some countries are now planning to monitor personal continuity in primary health care.^43^ A small improvement in personal continuity in the current review, such as a 10% increase in the index, had a significant impact on healthcare utilisation. In the studies in this review, continuity measures at the patient level were used. These measures are appropriate for scientific purposes, but physician-level measurements are needed in order for improvements in the organisation of healthcare centres to be implemented. Pereira Gray *et al* state: *'For a continuity measurement method to be useful, it needs to be simple for practices to use, to be easily understood by GPs and managers, and to capture meaningful continuity, ideally within a reasonably short timescale.'* ^44^ In some countries, primary care is performed by teams with different staff categories, which will affect measurements of personal continuity.^45^

In conclusion, these results show that higher, compared with lower, continuity in the relationship between GP and patients in ordinary primary care populations is associated with reduced mortality, admissions to hospital, and emergency department visits. Given that relatively small improvements in personal continuity, which may be achieved in most practices, significantly reduce healthcare consumption, and thus influence access to care, these findings have implications for healthcare policy.

## References

[1] Reid R, Haggerty J, McKendry R (2002). Defusing the confusion: concepts and measures of continuity of health care. Final report.

[2] van Walraven C, Oake N, Jennings A, Forster AJ (2010). The association between continuity of care and outcomes: a systematic and critical review. J Eval Clin Pract.

[3] Gray DJ, Sidaway-Lee K, White E (2018). Continuity of care with doctors - a matter of life and death? a systematic review of continuity of care and mortality. BMJ Open.

[4] Saultz JW, Lochner J (2005). Interpersonal continuity of care and care outcomes: a critical review. Ann Fam Med.

[5] Dyer SM, Suen J, Williams H (2022). Impact of relational continuity of primary care in aged care: a systematic review. BMC Geriatr.

[6] Baker R, Freeman GK, Haggerty JL (2020). Primary medical care continuity and patient mortality: a systematic review. Br J Gen Pract.

[7] Cabana MD, Jee SH (2004). Does continuity of care improve patient outcomes?. J Fam Pract.

[8] Page MJ, McKenzie JE, Bossuyt PM (2021). The PRISMA 2020 statement: an updated guideline for reporting systematic reviews. BMJ.

[9] Ryan RE, McKenzie JB, Thomson HJ, Higgins JT, Chandler J, Cumpston M (2022). Cochrane Handbook for Systematic Reviews of Interventions. Version 6.3.

[10] Ouzzani M, Hammady H, Fedorowicz Z, Elmagarmid A (2016). Rayyan - a web and mobile app for systematic reviews. Syst Rev.

[11] Sterne JA, Hernán MA, Reeves BC (2016). ROBINS-i: a tool for assessing risk of bias in non-randomised studies of interventions. BMJ.

[12] Guyatt G, Siemieniuk R What is GRADE?. https://dev-bestpractice.bmjgroup.com/info/toolkit/learn-ebm/what-is-grade.

[13] Tran B, Falster M, Jorm L (2018). Claims-based measures of continuity of care have non-linear associations with health: data linkage study. Int J Popul Data Sci.

[14] Ionescu-Ittu R, McCusker J, Ciampi A (2007). Continuity of primary care and emergency department utilization among elderly people. CMAJ.

[15] McCusker J, Tousignant P, Da Silva RB (2012). Factors predicting patient use of the emergency department: a retrospective cohort study. CMAJ.

[16] Menec VH, Sirski M, Attawar D (2005). Does continuity of care matter in a universally insured population?. Health Serv Res.

[17] Menec VH, Sirski M, Attawar D, Katz A (2006). Does continuity of care with a family physician reduce hospitalizations among older adults?. J Health Serv Res Policy.

[18] Leleu H, Minvielle E (2013). Relationship between longitudinal continuity of primary care and likelihood of death: analysis of national insurance data. PLoS One.

[19] Wensing M, Szecsenyi J, Laux G (2021). Continuity in general practice and hospitalization patterns: an observational study. BMC Fam Pract.

[20] Barker I, Steventon A, Deeny SR (2017). Association between continuity of care in general practice and hospital admissions for ambulatory care sensitive conditions: cross sectional study of routinely collected, person level data. BMJ.

[21] Tammes P, Purdy S, Salisbury C (2017). Continuity of primary care and emergency hospital admissions among older patients in england. Ann Fam Med.

[22] Hansen AH, Halvorsen PA, Aaraas IJ, Førde OH (2013). Continuity of GP care is related to reduced specialist healthcare use: a cross-sectional survey. Br J Gen Pract.

[23] Hetlevik Ø, Holmås TH, Monstad K (2021). Continuity of care, measurement and association with hospital admission and mortality: a registry-based longitudinal cohort study. BMJ Open.

[24] Sandvik H, Hetlevik Ø, Blinkenberg J, Hunskaar S (2022). Continuity in general practice as predictor of mortality, acute hospitalisation, and use of out-of-hours care: a registry-based observational study in norway. Br J Gen Pract.

[25] Engström S, Borgqúist L, Nordvall D (2019). [Personal physician continuity in primary care associated with fewer emergency room visits]. [Article in Swedish]. Lakartidningen.

[26] Kohnke H, Zielinski A (2017). Association between continuity of care in swedish primary care and emergency services utilisation: a population-based cross-sectional study. Scand J Prim Health Care.

[27] Maarsingh OR, Henry Y, van de Ven PM, Deeg DJ (2016). Continuity of care in primary care and association with survival in older people: a 17-year prospective cohort study. Br J Gen Pract.

[28] Nyweide DJ, Anthony DL, Bynum JPW (2013). Continuity of care and the risk of preventable hospitalization in older adults. JAMA Intern Med.

[29] Romaire MA, Haber SG, Wensky SG, McCall N (2014). Primary care and specialty providers: an assessment of continuity of care, utilization, and expenditures. Med Care.

[30] Yang Z, Ganguli I, Davis C (2022). Physician- versus practice-level primary care continuity and association with outcomes in medicare beneficiaries. Health Serv Res.

[31] Jee SH, Cabana MD (2006). Indices for continuity of care: a systematic review of the literature. Med Care Res Rev.

[32] Charlson ME, Pompei P, Ales KL, MacKenzie CR (1987). A new method of classifying prognostic comorbidity in longitudinal studies: development and validation. J Chronic Dis.

[33] Starfield B, Weiner J, Mumford L, Steinwachs D (1991). Ambulatory care groups: a categorization of diagnoses for research and management. Health Serv Res.

[34] Sidaway-Lee K, Pereira Gray OBE D, Harding A, Evans P (2021). What mechanisms could link GP relational continuity to patient outcomes?. Br J Gen Pract.

[35] Derksen F, Bensing J, Lagro-Janssen A (2013). Effectiveness of empathy in general practice: a systematic review. Br J Gen Pract.

[36] Howie JG, Heaney DJ, Maxwell M (1999). Quality at general practice consultations: cross sectional survey. BMJ.

[37] Ridd MJ, Lewis G, Peters TJ, Salisbury C (2011). Patient-doctor depth-of-relationship scale: development and validation. Ann Fam Med.

[38] Hjortdahl P (1992). The influence of general practitioners’ knowledge about their patients on the clinical decision-making process. Scand J Prim Health Care.

[39] Katz DA, McCoy K, Sarrazin MV (2014). Does improved continuity of primary care affect clinician-patient communication in VA?. J Gen Intern Med.

[40] Hjortdahl PER (1992). Continuity of care: general practitioners’ knowledge about, and sense of responsibility toward their patients. Fam Pract.

[41] Mercer SW, Jani BD, Maxwell M (2012). Patient enablement requires physician empathy: a cross-sectional study of general practice consultations in areas of high and low socioeconomic deprivation in scotland. BMC Fam Pract.

[42] Rodriguez HP, Rogers WH, Marshall RE, Safran DG (2007). The effects of primary care physician visit continuity on patients’ experiences with care. J Gen Intern Med.

[43] Fraser C, Clark G (2023). Measuring continuity of care in general practice. https://www.health.org.uk/reports-and-analysis/briefings/measuring-continuity-of-care-in-general-practice.

[44] Gray DP, Sidaway-Lee K, Whitaker P, Evans P (2023). Which methods are most practicable for measuring continuity within general practices?. Br J Gen Pract.

[45] Groot LJ, Janssen E, Westerman MJ (2025). Improving personal continuity in general practice: a focus group study. Br J Gen Pract.

