# Supplementary material for: Personal GP continuity improves healthcare outcomes in primary care populations: a systematic review
Source: Br J Gen Pract. 2025 Jul 14;75(757):e518–25. doi: 10.3399/BJGP.2024.0568 (PMC12729051; doi:10.3399/BJGP.2024.0568)
Supplement: Supplementary file 1 — Supplementary Information [file bjgp-75-757-0568-suppl.pdf]

Supplementary Information S1 - Search strategy

Databases searched: Embase, Medline, Scopus, Web of Science

<u>Search Strings</u>

EMBASE: November 2, 2023

(‘primary health care’/exp OR ‘primary care’ OR (general NEXT/1 pract*) OR family:ad OR (family NEXT/1 pract*) OR (family NEXT/1 physician*):de,it,lnk,ab,ti)

AND

(physicians/exp OR physician*:ti,ab OR practitioner*:ti,ab OR doctor*:ti,ab OR clinician*:ti,ab)

AND

('continuity effect'/exp OR continuity:ti,ab OR continuum:ti,ab OR (longitudinal* NEAR/3 (care OR healthcare)):ti,ab OR 'usual source':ti,ab OR 'regular source':ti,ab OR ((discontinu* OR inconsisten* OR fragment*) NEAR/3 (care OR healthcare)):ti,ab OR ((usual OR regular) NEAR/3 (physician* OR doctor* OR practitioner* OR clinician*)):ti,ab)

AND

((('clinical trial'/exp OR trial*:ti,ab OR 'randomization'/exp OR 'single blind procedure'/de OR 'double blind procedure'/de OR 'triple blind procedure'/de OR 'crossover procedure'/de OR 'placebo'/de OR randomi*:ti,ab OR rct:ti,ab OR ((random* NEAR/2 allocat*):ti,ab) OR 'single blind*':ti,ab OR 'double blind*':ti,ab OR (((treble OR triple) NEAR/1 blind*):ti,ab) OR 'crossover procedure':ti,ab OR placebo*:ti,ab OR 'prospective study'/exp OR 'prospective study':ti,ab) NOT ('case study'/de OR 'case report':ti,ab OR 'abstract report'/de OR 'letter'/de OR editorial:pt OR letter:pt OR note:pt))

OR

("clinical study"/de OR “controlled study”/de OR "case control study"/de OR "family study"/de OR "longitudinal study"/de OR "retrospective study"/de OR "cohort analysis"/de OR "observational study"/de OR “controlled stud*”:ti,ab OR “cohort stud*”:ti,ab OR "cohort analy*":ti,ab OR "case control stud*”:ti,ab OR longitudinal:ti,ab OR retrospective:ti,ab OR “follow-up stud*”:ti,ab OR “observational stud*”:ti,ab OR “epidemiologic* stud*”:ti,ab OR "cross-sectional stud*”:ti,ab OR "cross sectional stud*”:ti,ab OR questionnaire/exp OR questionnaire*:ti,ab OR survey*:ti,ab))

AND

([danish]/lim OR [english]/lim OR [norwegian]/lim OR [swedish]/lim)

AND

[2000-2024]/py

NOT

([conference abstract]/lim OR [conference paper]/lim OR [conference review]/lim)

Pubmed Medline: November 1, 2023

(“primary health care”[mh] OR "primary care"[all fields] OR "physicians, family"[mh] OR “general pract*”[all fields] OR "family"[ad] OR “family pract*”[all fields] OR “family physician*”[tw])

AND

(physicians[mh] OR physician*[tiab] OR practitioner*[tiab] OR doctor*[tiab] OR clinician*[tiab])

AND

(“continuity of patient care”[mh:noexp] OR continuity[tiab] OR continuum[tiab] OR "longitudinal care"[tiab:~3] OR "longitudinal healthcare"[tiab:~3] OR "usual source"[tiab] OR "regular source"[tiab] OR “discontinuity care”[tiab:~3] OR “inconsistent care”[tiab:~3] OR “fragmented care”[tiab:~3] OR “fragmentation care”[tiab:~3] OR “discontinuity healthcare”[tiab:~3] OR “inconsistent healthcare”[tiab:~3] OR “fragmented healthcare”[tiab:~3] OR “fragmentation healthcare”[tiab:~3] OR "usual physician"[tiab:~3] OR "usual doctor"[tiab:~3] OR "usual practitioner"[tiab:~3] OR "usual clinician"[tiab:~3] OR "regular physician"[tiab:~3] OR "regular doctor"[tiab:~3] OR "regular practitioner"[tiab:~3] OR "regular clinician"[tiab:~3]OR "usual physicians"[tiab:~3] OR "usual doctors"[tiab:~3] OR "usual practitioners"[tiab:~3] OR "usual clinicians"[tiab:~3] OR "regular physicians"[tiab:~3] OR "regular doctors"[tiab:~3] OR "regular practitioners"[tiab:~3] OR "regular clinicians"[tiab:~3])

AND

((("clinical trial"[publication type] OR “clinical trials as topic”[mh] OR "randomized"[tiab] OR "placebo"[tiab] OR "randomly"[tiab] OR "trial"[ti]) NOT ("animals"[mh] NOT "humans"[mh]))

OR

("epidemiologic studies"[mh:noexp] OR "case control studies"[mh] OR "cohort studies"[mh] OR "cross-sectional studies"[mh:noexp] OR "case control"[tiab] OR "cohort stud*"[tiab] OR "cohort analy*"[tiab] OR "follow-up stud*"[tiab] OR "observational stud*"[tiab] OR longitudinal[tiab] OR retrospective[tiab] OR "cross sectional"[tiab] OR "controlled stud*"[tiab] OR “observational studies as topic”[mh] OR "observational study”[publication type] OR “surveys and questionnaires”[mh] OR questionnaire*[tiab] OR survey*[tiab]))

AND

(swedish[language] OR danish[language] OR norwegian[language] OR english[language])

AND

("2000"[Date - Publication] : "2024"[Date - Publication])

And limits to languages English, Swedish, Norwegian or Danish

Scopus: November 1, 2023

((TITLE-ABS-KEY (“primary health care”)) OR (TITLE-ABS-KEY (“primary care”)) OR (TITLE-ABS-KEY (“general pract*”)) OR (AFFIL (family)) OR (TITLE-ABS-KEY (“family pract*”)) OR (TITLE-ABS-KEY (“family physician*”)))

AND

(TITLE-ABS-KEY (physician* OR practitioner* OR doctor* OR clinician*))

AND

((TITLE-ABS-KEY (continuity OR continuum)) OR (TITLE-ABS-KEY ((longitudinal*) W/3 (care OR healthcare))) OR (TITLE-ABS-KEY ("usual source" OR "regular source")) OR (TITLE-ABS-KEY ((discontinu* OR fragment* OR inconsisten*) W/3 (care OR healthcare))) OR (TITLE-ABS-KEY ((usual OR regular) W/3 (physician* OR practitioner* OR doctor* OR clinician*))))

AND

((TITLE-ABS-KEY(trial* OR random* OR placebo OR “single blind*” OR “double blind*” OR “triple blind*” OR “treble blind*” OR “crossover*” OR “cross-over*” OR “prospective stud*”))

OR

(TITLE-ABS-KEY(“epidemiologic* stud*” OR “case control” OR “cohort stud*” OR “cohort analy*” OR “cross sectional” OR “follow-up stud*” OR “observational stud*” OR longitudinal OR retrospective OR “controlled stud*” OR questionnaire* OR survey*)))

AND

(LIMIT-TO (LANGUAGE , "English") OR LIMIT-TO (LANGUAGE , "Norwegian") OR LIMIT-TO (LANGUAGE , "Swedish") OR LIMIT-TO (LANGUAGE , "Danish"))

AND

(PUBYEAR AFT 1999)

AND NOT

(DOCTYPE(CP) OR DOCTYPE(CR))

Web of Science: November 2, 2023

((TS=(“primary health care”)) OR (TS=(“primary care”)) OR (TS=(“general pract*”)) OR (AD=(family)) OR (TS=(“family pract*”)) OR (TS=(“family physician*”)))

AND

(TS=(physician* OR practitioner* OR doctor* OR clinician*))

AND

((TS=(continuity OR continuum)) OR (TS=((longitudinal*) NEAR/3 (care OR healthcare))) OR (TS=("usual source" OR "regular source")) OR (TS=((discontinu* OR fragment* OR inconsisten*) NEAR/3 (care OR healthcare))) OR (TS=((usual OR regular) NEAR/3 (physician* OR practitioner* OR doctor* OR clinician*))))

AND

((TS=(trial* OR random* OR placebo OR “single blind*” OR “double blind*” OR “triple blind*” OR “treble blind*” OR “crossover*” OR “cross-over*” OR “prospective stud*”))

OR

(TS=(“epidemiologic* stud*” OR “case control” OR “cohort stud*” OR “cohort analy*” OR “cross sectional” OR “follow-up stud*” OR “observational stud*” OR longitudinal OR retrospective OR “controlled stud*” OR questionnaire* OR survey*)))

AND

(PY=2000-2024)

And limits to languages English, Swedish, Norwegian or Danish

Table S1. Summary of the characteristics and outcomes of the included studies.

Abbreviations of continuity measures: UPC= Usual Provider of Care Index, B&B = Bice and Boxerman Continuity Index, HH = Herfindahl–Hirschman Index.

Physician speciality: GP = general practitioner, FP = family physician, NP= nurse practitioner, PA = physician assistant, PCP = primary-care physician working as a generalist

Outcome(s): ACSC = ambulatory care-sensitive conditions

Confounders: 1. age; 2. gender; 3. marital status; 4. income; 5. education; 6. comorbidity; 7. GP visits; 8. emergency-department visits; 9. hospitalisations; 10. self-rated health/function, ( ) measure at the group level

**Author**<br>**Year**<br>**Country**	**Study type**<br>**Data period**<br>**Length of outcome period (months)**	**Number of patients**<br>**Age**	**Measure of continuity**<br>Physician speciality<br>*Length of continuity*<br>*Preceded outcome period (Yes/No)*	**Outcome(s)**<br>**Type(s) of analysis**	**Reported results**	**Overall risk of bias**	**Confounders considered**
Barker et al.<br>2016<br>England	Retrospective cohort<br>2011–2013<br>24	n = 230 472<br>62–82 years	UPC<br>GP<br>24 months<br>*No*	Hospitalisation for ACSC<br>Multivariable linear regression	High (< 0.7) vs low (< 0.4) continuity: 12.5 % (95% CI 9.45–19.29) fewer hospitalisation for ACSC	Moderate	1, 2, (4), 6, 7, referrals to specialist care
Engström et al.<br>2019<br>Sweden	Retrospective cohort<br>2015–2016<br>12	n = 122 400<br>All ages	B&B<br>GP<br>24 months<br>*No*	Emergency-department visits<br>Multivariable linear regression	The 15% of patients with the highest continuity had 49% fewer emergency-department visits than the 15% with the lowest continuity.<br>Regression coefficient for B&B -0.107. p < 0.001	Moderate	1, 2, 3, 4, 5, 6, 7, distance to hospital
Hansen et al.<br>2013<br>Norway	Retrospective questionnaire data from the sixth Tromsø Study<br>2007–2008<br>14	n = 8 611<br>30–87 years	Duration, in years, of the patient-GP relationship<br>GP<br>>2 years<br>*Yes*	Hospitalisations<br>Logistic regression	Patient-GP relationship of more than 2 years vs. less than 2 years.<br>OR 0.76 (95% CI = 0.64–0.90).	Moderate	1, 2, 3, 4, 6,10
Hetlevik et al.<br>2021<br>Norway	Registry-based, longitudinal cohort study<br>Continuity 2016–2017<br>Outcome 2018<br>12	n = 757 873<br>60–90 years	UPC<br>GP<br>24 months<br>*Yes*	Mortality<br>Hospitalisations<br>Logistic regression<br>Multivariable regression	An UPC index increase by 0.2<br>Mortality: -8.3%<br>Hospitalisations: -5.8%<br>Hospitalisation for ACSC: -8.1% (95% CI 7.1- 9.1)<br>p < 0.001 for all	Moderate	1, 2, 4, 5, 7, 9

Ionescu et al.<br><br>2007<br><br>Canada	Cross-sectional study<br><br>2000–2001<br><br>24	n = 95 173<br><br>≥ 65 years	UPC<br><br>GP*<br>*79 % GPs, the rest specialists<br><br>24 months<br>*No*	Emergency-department visits<br><br>Poisson regression analysis	UPC low (≤ 0.5) vs. high (> 0.8)<br>RR 1.46 (95% CI 1.44–1.48)<br>Medium (> 0.5 and ≤ 0.8) vs. high (> 0.8)<br>RR 1.27 (95% CI 1.25–1.29)	Moderate	1, 2, (4), 6, 7, distance to emergency department
Kohnke et al.<br><br>2017<br><br>Sweden	Population-based cross-sectional study<br><br>2012–2014<br><br>36	n = 8 185<br><br>All ages	B&B, UPC<br><br>GP<br><br>36 months<br>*No*	ES visits = visits to GP out of hours + emergency-department visits<br><br>Negative binominal regression	B&B low (0.0) vs. high (1.0)<br>RR 0.6 (95% CI 0.73–0.47)	Moderate	1, 2, 6, 7
Leleu et al.<br><br>2013<br><br>France	Population-based cross-sectional study<br><br>2007–2010<br><br>36	n = 325 742<br>All ages	B&B<br><br>GP<br><br>36 months<br>*No*	Mortality<br><br>Cox proportional hazards survival regression	A 0.1 increase in B&B<br>HR 0.96 (95% CI 0.95–0.96)<br>p < 0.0001	Moderate	1, 2, 4, 6
Maarsingh et al.<br><br>2016<br><br>The Netherlands	Retrospective cohort study<br>1992–2009<br><br>1999-2013	n = 1 708<br><br>≥ 60 years	HH<br><br>GP<br><br>*17 years*<br>*Yes*	Mortality<br><br>Cox regression analysis	HH low (≤ 0.5) vs. high (1,0)<br>HR 1.20 (95% CI 1.01–1.42)<br>HH moderate (0.50–0.56) vs. high (1.0)<br>HR 1.17 (95% CI 0.98–1.41)	Moderate	1, 2, 3, (4), 6, 7, 10, smoking, alcohol use
McCusker et al.<br><br>2012<br><br>Canada	Retrospective cohort study<br><br>2003—2006<br><br>12	n = 271 990<br><br>≥ 18 years	UPC<br><br>FP<br><br>24 months<br>*Yes*	Emergency-department visits<br><br>Binomial regression	UPC low (≤ 0.4) vs. high (≥ 0.80)<br>IRR 1.00 (95% CI 0.97–1.03)<br>UPC medium (0.40–0.79) vs. high (≥ 0.8)<br>IRR 1.00 (95% CI 0.97–1.02)	Moderate	1, 2, 3, (4), (5), 6, 7, 8, 9
Menec et al.<br><br>2005<br><br>Canada	Retrospective cohort study<br><br>1998–1999<br><br>24	n = 536 893<br><br>All ages	UPC<br><br>FP<br><br>24 months<br>*No*	Emergency-department visits<br><br>Multivariable logistic regression	UPC low (≤ 0.5) vs. high (> 0.50)<br>0–14 years OR 0.91 (95% CI 0.87–0.94)<br>15+ years OR 0.90 (95% CI 0.87–0.92)	Moderate	1, 2, 3, (4), 6, 7
Menec et al.<br><br>2006	Retrospective cohort study<br><br>1990–1991	n = 1 863<br><br>≥ 66 years	UPC cut-off limit at 75%. Above = high, below = low<br><br>FP	Hospitalisation for ACSC	UPC high (≥ 0.75) vs. low (< 0.75)<br>OR 0.67 (95% CI 0.51–0.90)	Moderate	1, 2, 3, 5, 6, 10

Canada	1996–1997<br><br>48		24 months<br>*No*	Multivariable logistic regression			
Nyweide et al.<br><br>2013<br><br>USA	Retrospective cohort study<br><br>2008–2010<br><br>24	n = 3 276 635<br><br>> 65 years	UPC, HH<br><br>PCP, NP, PA*<br><br>24 months<br>*No*	Hospitalisation for ACSC<br><br>Cox proportional hazards regression	A 0.1 increase in UPC<br>HR 0.98 (95% CI 0.98–0.98)	Moderate	1, 2, (4), (5), 6, 7, 9<br><br>*Some visits were with nonphysician providers, such as nurse practitioners or physician assistants.
Romaire et al.<br><br>2014<br><br>USA	Cross-sectional study<br><br>2007–2009<br><br>12	n = 461 441<br><br>> 65 years	B&B, UPC<br><br>PCP*<br><br>12 months<br>*Yes*	Hospitalisations<br><br>Emergency-department visits<br><br>Multivariate, negative binomial regression	B&B High (> 0.53) vs. low (< 0.29)<br>Hospitalisations<br>IRR 0.91 (95% CI 0.90–0.93)<br>Emergency-department visits<br>IRR 0.85 (95% CI 0.84–0.86)	Moderate	1, 2, (4), (5), 6<br><br>*The study covered both generalists and specialists. We report population and results for generalists in primary care
Sandvik et al.<br><br>2021<br><br>Norway	Registry-based observational<br><br>2009–14<br><br>12	n = 4 552 978<br><br>All ages	Duration, in years, of the patient-GP relationship<br><br>GP<br><br>1 year to > 15 years<br>*Yes*	Mortality<br>Emergency hospitalisations<br><br>Multiple logistic regression	Patient-GP relationships,<br>2–3 years vs. 1 year<br>Death OR 0.92 (95% CI 0.86–0.98)<br>Emergency hospitalisation<br>OR 0.88 (95% CI 0.86–0.90)<br><br>Patient-GP relationships,<br>>15 years vs. 1 year<br>Death OR 0.75 (95% CI 0.70–0.80)<br>Emergency hospitalisation<br>OR 0.72 (95% CI 0.70–0.73)	Moderate	1, 2, 6, 7, 9, ethnicity
Tammes<br><br>2017<br><br>England	Prospective cohort<br><br>2012–14<br><br>24	n = 8 248<br><br>≥ 65 years	B&B<br><br>GP<br><br>24 months<br>*No*	Emergency hospitalisations<br><br>Mixed effects Weibull regression	B&B low (< 0.25) vs. high (1.00)<br>OR 1.12 (95% CI 0.88–1.43)<br>B&B middle (0.25-0.38) vs. high (1.00)<br>OR 1.05 (95% CI 0.83 –1.33)	Moderate	1, 2, (4), 6, 7, 9
Tran et al.<br><br>2018<br><br>Australia	Prospective cohort<br><br>2006–2009<br><br>12	n = 222 943<br><br>≥ 45 years	B&B, UPC<br><br>GP<br><br>24 months<br>*Yes*	Emergency hospitalisations<br><br>Multivariable logistic regression	B&B high (1.00) vs. low (< 0.50)<br>OR 0.87 (95% CI 0.83–0.91)<br>medium (0.75–0.99) vs. low (< 0.50)<br>OR 1.03 (95% CI 0.99–1.07)	Moderate	1, 2, 3, 4, 6, 9, 10

Wensing et al.<br>2021<br>Germany	Prospective cohort<br>2017<br>12	n= 1 760 202<br>> 18 years	UPC, HH<br>GP<br>12 months<br>*No*	Hospitalisations<br>Linear mixed models with Generalised Estimating Equations	An UPC index increase by 0.1<br>Hospitalisations<br>-9.74 % (95% CI -9.94- 9.54)	Moderate	1, 2, 6, living in nursing home
Yang et al.<br>2022<br>USA	Retrospective Cohort<br>2011–2017	n = 2 359 400<br>≥ 65 years	B&B<br>PCP<br>12 months<br>*Yes*	Emergency-department visits<br>Hospitalisation for ACSC<br>Ordinary least square regression	B&B index increase by 0.1<br>Hospitalisations -10.2%*<br>Emergency department visits -13.4%*	Moderate	1, 2, 3, 6, ethnicity<br>*An increase in the B&B score in the previous year of 0.1 was associated with 19 fewer than the initial 187 hospitalisations and 44 fewer than the initial 329 emergency-department-visits, both per 1000 patients. We have converted these numbers to percentages.
